# Design and Manufacturing of an Ultra-Low-Cost Custom Torque Sensor for Robotics

**DOI:** 10.3390/s18061786

**Published:** 2018-06-01

**Authors:** Rodrigo Pérez Ubeda, Santiago C. Gutiérrez Rubert, Ranko Zotovic Stanisic, Ángel Perles Ivars

**Affiliations:** 1 Department of Mechanical and Materials Engineering, Universitat Politècnica de València, Valencia 46022, Spain; scgutier@mcm.upv.es; 2 Department of Systems Engineering and Automation, Universitat Politècnica de València, Valencia 46022, Spain; rzotovic@isa.upv.es; 3 Department of Computer Systems and Computation, Universitat Politècnica de València, Valencia 46022, Spain; aperles@disca.upv.es

**Keywords:** torque sensor, design of sensors, manufacturing of low cost sensors

## Abstract

This article describes a new, very low-cost torque sensor. It was designed to obtain a geometric shape suitable for very affordable manufacturing by machining. The torque sensor was developed under the principle of measurement by strain gauges. It has been designed in order to make manufacturing operations as simple as possible. Optimization was achieved through finite element analysis. Three test sensors for 1, 5, and 20 Nm were designed and machined. Calibration of the three sensors has been carried out obtaining excellent results. An analysis of the dimensional quality of the product and associated costs demonstrates that manufacturing is possible with very simple machining operations, standard tools, and economic equipment.

## 1. Introduction

Robot arms have been used in industry for more than five decades in applications such as automotive, electronics, and shipbuilding [1,2]. The “classic” robot arms are not oriented for interaction with humans. Nevertheless, the new generations of robots, like collaborative [3], lightweight, exoskeletons, do make controlled contact with humans. This requires modifications in their design in order to include devices that allow for this. For these applications, it is very useful to be able to measure forces and torques. The possibility to measure and control these variables increases the applicability of robots, enabling improved operation and performance while enhancing the safety of their respective activities [2,3,4,5]. In addition, providing precise torque values is a key factor for dynamic decoupling and control of the force-movement of robotic arms [6].

Thus, measurement and estimation of force and torque are key components of a collaborative robot. The expansion of robotic applications, the growth of new demands, and the high cost of commercial sensors have brought an active development of force and torque sensors, with different configurations and applications [1,7].

Commercial six degrees of freedom (6-DOF) force/torque sensors may cost more than €4000 [8,9], and those for torque only (one degree of freedom) are between €1400 and €3000 [10,11]. However, the use of the latter ends up being more expensive since more units are needed in the robot.

While current sensors offer good characteristics, they are not simple to manufacture, an issue that has yet to be addressed despite having been considered as one of the primary design requirements detailed by Hirzinger et al. [12] in their first work on force/torque sensors.

An analysis of the costs of the other components that do not comprise the elastic element indicates that these are very economic, with a general cost of less than €100. This indicates that the process of manufacturing complex geometries for elastic elements raises the production costs of the sensor. Due to this reason, the objective of this project was to develop a uniaxial sensor, designed via favorable geometry, from the point of view of manufacture by machining in order to reduce costs as much as possible while maintaining the performance.

**Figure 1:**
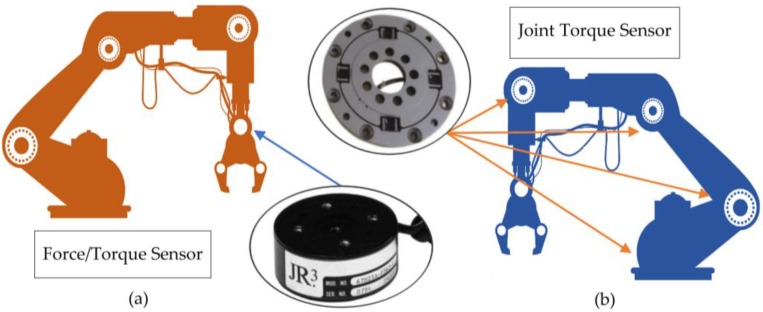
Use of sensors in a robotic arm, (**a**) single force and torque sensor located in the manipulator, (**b**) torque sensors arranged in each joint of the robot arm.

In general, two types of sensors may be found inside a robot—those that are located at the end effector (Figure 1a), which are complex sensors comprising six force and torque components, and those located in the joints of the robot (Figure 1b), which are purely torsional sensors [2]. The latter, being integrated in the joints, enable application of advanced control methods for joint torque control, vibration damping, and stiffness control [13]. Torque sensors are widely used in arms intended for assistance for the disabled [14] and since customized, economical designs are required, custom manufacturing is necessary in order to obtain the desired characteristics.

From the point of view of application, force/torque sensors are used in industry to control the interaction force between the robot and the environment for tasks such as polishing, deburring, engraving, etc.

Force/torque sensors are usually comprised of six components (6-DOF) and require relatively complex geometries, which complicates manufacturing and results in increased costs. On the other hand, their main limitation is that they detect force/torque in the end effector.

In contrast, a lightweight and collaborative robot must be able to react if any of its links come into contact with an unexpected object. For this reason, it is necessary that each joint is equipped with its own sensor. However, these may be torque sensors with one degree of freedom and may be designed with much simpler and more economical geometries.

**Figure 2:**
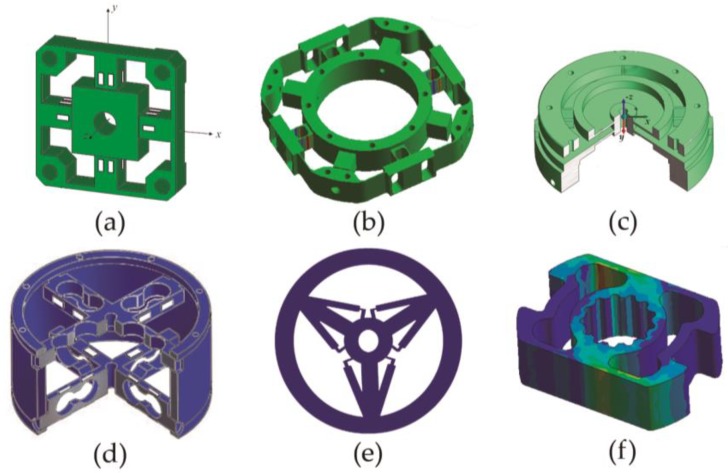
Types of force/torque sensor structures: (**a**) Crossbeam, (**b**) Crossbeam modified, (**c**) Body E-type membrane (EE) (**d**) Sliding structure, (**e**) Four-bar linkage shape, (**f**) Square cube.

As an example, some force/torque and torque-only sensors are shown in Figure 2. The work of Ma and Song [15] (Figure 2a) represents the typical 6-DOF force/torque, consisting of an elastic body containing crossbeams.

Sun et al. [4] developed a 6-DOF sensor for a spatial robot used in the Chinese space station. Its elastic body with crossbeams is shown in Figure 2b. It has slim beams not completely solid, with cavities, as main feature. This allows to concentrate the stresses to obtain better sensitivity.

Liang et al. [16] proposed the design of a sensor based on a more complex geometry called E-type membrane, or EE (Figure 2c). This sensor enables the measurement of the six components of force and torque with a low degree of coupling, but with the great disadvantage of requiring a laborious and complex design and, therefore, high manufacturing cost.

Wu and Cai [17] developed a more complex 6-DOF sensor by assembling three different parts. They called this design “Sliding structure”. The sensor may be appreciated in Figure 2d. It has two independent elastic elements, assembled to the external ring through a top and a bottom cover. A better decoupling is obtained by dividing the measurement between two elastic elements.

Zhang et al. [2] proposed a design of a torque sensor based on the crossbeams type, but with a four-bar link shape (see Figure 2e). It has high sensitivity without loss of stiffness. Despite these good features, their design is very complex to manufacture causing an increase of the price.

Figure 2f represents another example of geometry used for a torque sensor, such as that detailed by Khan et al. [18], presenting a design called Square Cube that greatly facilitates the installation of strain gauges. However, it increases the complexity of the elastic element, as well as the manufacturing costs.

The previous examples show how the geometry of the elastic body is becoming more and more complex in order to obtain a better performance of the sensor. This article proposes a simple geometry uniaxial sensor allowing for the reduction of manufacturing costs.

The article is structured as follows: Section 2 presents the design methodology, materials, and analysis of design and manufacturing requirements; Section 3 shows the results and discussion of the design and manufacture of the sensor, corresponding calibration, and cost analysis; finally, the conclusions are presented in Section 4.

## 2. Design Methodology

The elastic element is the most expensive part, having to comply with the specifications of torque and dimensions required for the target application. For that reason and before this manufacturing, its behavior was validated through finite element analysis in SolidWorks Simulation (Computer Aided Engineering, CAE, application).

To obtain an economic sensor, complex geometries which require elaborate cutting operations and special tools must be avoided since they increase manufacturing time and require numerical control programs for machine tools capable of achieving this level of complexity.

Bearing in mind the ease of machining and understanding that the value of dimensional and geometric tolerances directly influences this concept, small variations have been introduced into the 3D models used in the CAE simulations in order to test how these variations affect the end functionality of the sensor. These include variations in dimension, position of elements, and geometrics (flatness, parallelism, coaxiality, etc.). In this way, it is possible to establish the level of precision required during the manufacturing process.

### 2.1. Selection of Material for Elastic Element

The material with which the elastic element of the sensor will be manufactured must comply with a series of characteristics, such as easy machining by chip removal, high sensitivity to applied torque, linear response deformation, low density, and fairly isotropic and homogeneous behavior.

For the elastic element, aluminum or steel alloys are usually used. An analysis of the characteristics of these materials indicates that the advantages of aluminum are better machinability and lower weight. In contrast, the advantages of steel are greater Young’s modulus and elastic limit.

In the articles referenced [1,2,19,20], different authors have used different aluminum alloys. After evaluating several proposals, it was decided to use 7075-T6 aluminum alloy. Table 1 shows its main properties.

This aluminum alloy has very good characteristics such as modulus of elasticity, high elastic limit, and very easy machining, comparable to the characteristics of some steels, which allows support of high tension and deformation levels while maintaining a good safety factor against overload.

Although the aluminum is more expensive than steel, the price difference is negligible for such small lightweight sensors. Finally, aluminum has been chosen as it presents a good combination of benefits and price.

Since one of the goals is to obtain a parametric design valid for sensors of various sizes (this report details testing for 1, 5, and 20 Nm), a maximum diameter of 65 mm and thickness of 10 mm were used as initial requirements. These dimensions are in strict accordance with available standard dimensions of raw materials—in this case, for cylindrical bars. Once again, this results in a reduction of costs. In addition, these values correspond to those acceptable for the real requirements of a collaborative robot arm prototype in which the sensors will be applied. It should be noted that the proposed method is valid for any other dimension or torque requirements.

### 2.2. Analysis of Design Requirements

**Figure 3:**
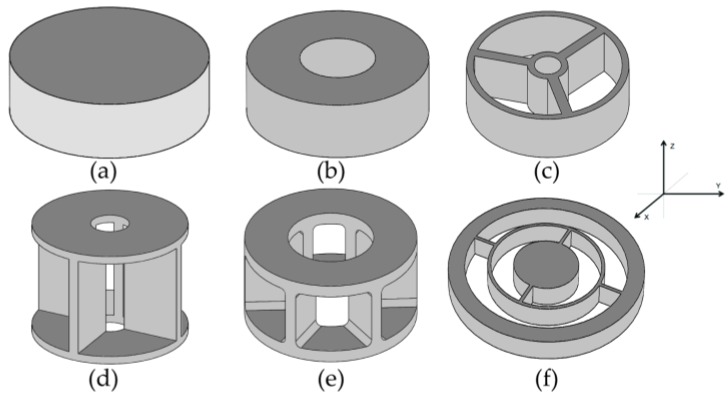
Types of torque sensor structures with gauges: (**a**) Solid Cylinder, (**b**) Hollow Cylinder, (**c**) Hub-Sprocket (**d**) Hollow Cruciform, (**e**) Hollow Hexaform, (**f**) Spoke Topology. Adapted from [2].

In the search for a single simple design for the elastic element of the sensor, the most common geometries were analyzed within the sensors that use strain gauges. Zhang et al. [2] and Aghili et al. [21] studied the most common types of elastic elements used in torque sensors, which may be seen in Figure 3.

The Solid and Hollow Cylinders (Figure 3a,b) are simple, rigid structures. However, they are sensitive to nontorsional components. Thus, designs have evolved to other types of more complex geometries that minimize this effect while greatly increasing sensor sensitivity.

The most common type of elastic element is similar to the Hub-Sprocket (Figure 3c) but with the inclusion of four beams or crossbars in its central body. This variant of the Hub-Sprocket is known as the Maltese Cross.

**Figure 4:**
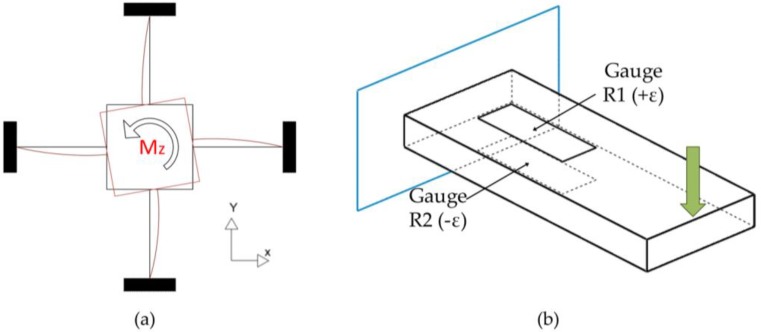
Hub-Sprocket Geometry: (**a**) Beam deformation; (**b**) Attachment of gauges.

Ma and Song [15], Kim G.S. et al. [22], and Kim Y.G. et al. [23] designed three- and six-component sensors using crossbeam geometry. These sensors have good rigidity but low isotropy and a high degree of coupling, which is why other researchers modified their characteristics to improve performance [19]. Although the Hub-Sprocket topology sensor has lower performance compared to more complex ones, such as the “E-type membrane”, its torque measurement is better since it enables greater sensitivity. This makes it one of the most commonly used geometries to measure torque component *M_z_* (see Figure 4a) and it is favored even more considering that its geometry is not one of the most complex, thus enabling easier manufacture.

The Hollow Cruciform design (Figure 3d) is used in commercial torque sensors. This sensor has good sensitivity, but the stiffness is low and nontorsional torques are high. The Hollow Hexaform sensor (Figure 3e) is similar in its basic geometry to the Hollow Cruciform. However, due to the increased number of wing pairs and the shorter height, the Hollow Hexaform sensor is stiffer and more sensitive. Finally, torque sensors with Spoke Topology (Figure 3f) provide high sensitivity but the lack of stiffness introduces a joint angle error.

Considering the characteristics observed, it was concluded that the development of a single simple geometry for the design of the sensor using the Hub-Sprocket typology could reduce manufacturing costs, thus obtaining an economic, lightweight, customized torque sensor (for a specified torque with required dimensions) that facilitates proper performance.

### 2.3. Selection and Arrangement of Strain Gauges

The Hub-Sprocket topology with four beams was selected, in which four gauges will be installed. Two gauges will be placed in one of the four beams (in each of its side walls) and the other two in the beam diametrically opposite. With this configuration, in each pair of gauges, one will work under compression and the other under tension. In this way, gain is multiplied by two and nonlinearity is reduced.

The Hub-Sprocket type geometry is characterized by deformation of its beams. Deformation acts in the same way as in a recessed beam (Figure 4a), enabling use of uniaxial gauges to measure torque, as shown in Figure 4b. Since there are four beams, two will be used to attach the four gauges, forming a complete Wheatstone bridge, which facilitates the best measurements [24].

The strain gauges used are models KFH-6-120-C1-11L1M2R and KFH-6-350-C1-11L1M2R, supplied by the company Omega [25], whose characteristics are summarized in Table 2. These uniaxial gauges have a cost of €8 per unit and good performance.

**Figure 5:**
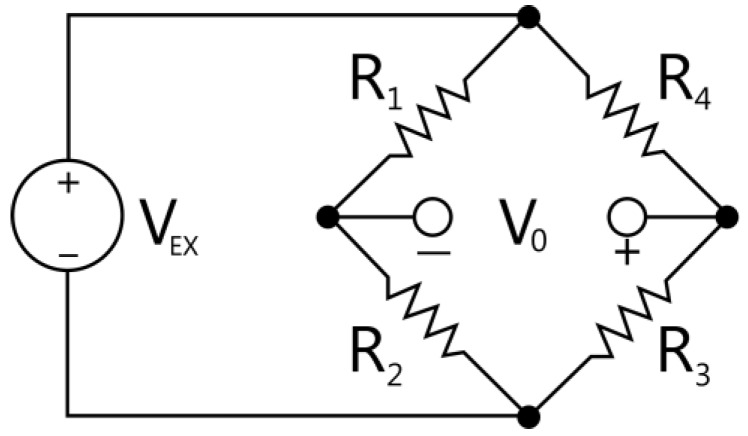
Complete Wheatstone bridge.

The arrangement of the four gauges corresponds to that of a complete Wheatstone bridge, as shown in Figure 5. 

The equation that defines the Wheatstone bridge output [24] is

(1) $$V_{0}=\left[\frac{R_{3}-\Delta R_{3}}{R_{3}-\Delta R_{3}+R_{4}+\Delta R_{4}}-\frac{R_{2}+\Delta R_{2}}{R_{1}-\Delta R_{1}+R_{2}+\Delta R_{2}}\right]V_{EX},$$

where $V_{EX}$ is input voltage and $R_{i}$ the resistances of the gauges. In an ideal sensor, all gauges are equal, the dimensions of the elastic element are perfect, and each gauge is placed at the site of maximum deformation of the element. In this case, all the values are symmetric and, therefore, $R_{1}=R_{2}=R_{3}=R_{4}=R$ and $\Delta R_{1}=\Delta R_{2}=\Delta R_{3}=\Delta R_{4}=\Delta R=R\Delta GF\Delta \varepsilon_{m}$. Where R is the resistance of the gauge, GF is the gauge factor, and $\varepsilon_{m}$ is the measured strain. The bridge output voltage obtained in this case is

(2) $$V_{0}=\left[-\frac{\Delta R}{R}\right]V_{EX}=-GF\cdot \varepsilon_{m}\cdot V_{EX},$$

Due to dimensional errors and the location of the gauges, the variations obtained by each will be different, so that $\Delta R_{1}\neq \Delta R_{2}\neq \Delta R_{3}\neq \Delta R_{4}$. In this case, the formula (1), expressed in accordance with strains, results as

(3) $$V_{0}=\left[\underset{A}{\underset{︸}{\frac{1}{2+GF\left(\varepsilon_{4}-\varepsilon_{3}\right)}}}-\underset{B}{\underset{︸}{\frac{GF}{2+GF\left(\varepsilon_{4}-\varepsilon_{3}\right)}}}\cdot \varepsilon_{3}-\underset{C}{\underset{︸}{\frac{1}{2+GF\left(\varepsilon_{2}-\varepsilon_{1}\right)}}}-\underset{D}{\underset{︸}{\frac{GF}{2+GF\left(\varepsilon_{2}-\varepsilon_{1}\right)}}}\cdot \varepsilon_{2}\right]V_{EX},$$

This expression has four addends, *A*, *B*, *C,* and *D,* with a difference of strains in the denominator. Since the strains in this study are of the order of thousandths, their difference will not generate important errors in linearity, as any difference would be of thousandths, thus when dividing 1 by this value, no significant numerical variation will result for the factors, *A*, *B*, *C*, and *D*. The values from the strain analysis in the direction of the X axis (see Section 3.2) are substituted into equation (3) and reach approximately 0.499 for factors *A* and *C*, which would be annulled analytically, and 0.999 for factors *B* and *D*—the latter being a common factor for the difference in strain $\left(\varepsilon_{3}-\varepsilon_{2}\right)$. As will be seen in later analyses, the greater effect on the bridge output result is influenced by this difference in strain. In the following sections, the influence of manufacturing variations on these strains shall be analyzed.

### 2.4. Manufacturing Requirements and Functional Verification

Since the elastic element is metallic and given that its construction will be unitary, or of very few equal elements, machining is the most viable option. However, it is usually an expensive process, since it is possible to generate almost any geometry coordinating simultaneously the movements of several axes of the machine. To reduce costs and avoid the use of expensive equipment, geometries are restricted to those that can be obtained with a single degree of freedom, i.e., cut feed movement of the tool along a single axis.

**Figure 6:**
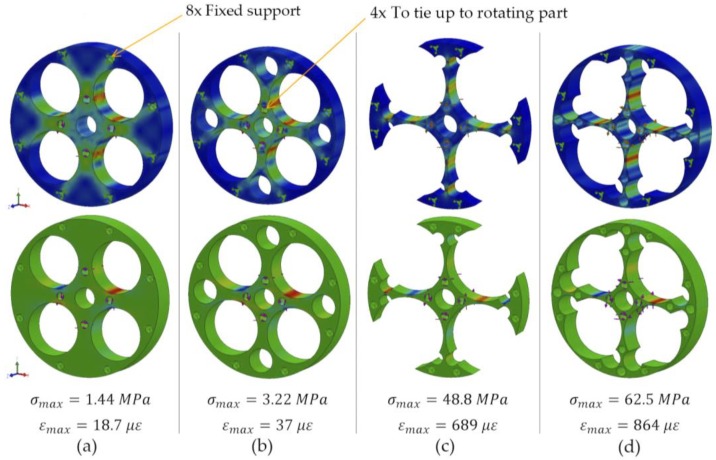
Example of different geometries, (**a**–**d**) with the results of CAE analysis.

Only those machining operations will be used in which the cut feed is along the Z axis of the machine, according to ISO-841, as in the case of drilling, reaming, threading, etc. Considering these restrictions, Figure 6 shows some of the geometric alternatives considered for the design of a 1 Nm sensor, with their corresponding finite element analyses to determine the value of deformations resulting in each of the proposals.

The analysis showed stresses (Figure 6, upper blue image) and strains (Figure 6, lower image) when loading the sensor through four central holes of small diameter, where it is connected to the joint axis, with a torque of 1 Nm along the Z axis. The body of the sensor is tied to the device, i.e. robot arm, using its 8 outermost holes of small diameter. The lower part of Figure 6 shows the maximum stresses obtained from each geometry when applying the resulting torque and the microstrains.

As can be seen in the results of Figure 6, design alternative (d) had the greatest strains when a torque of 1 Nm was applied. The main characteristic of this alternative is the hollowing of the structure by means of four drills with a diameter of 23.5 mm, which comprise the four crossbeams (Hub-Sprocket geometry). Gauges shall be installed in areas of maximum deformation: red for tension and blue for compression. The way to pass the required wires will be through the central hole. The curved area, where the gauges will be attached, is compatible with the minimum radius of curvature allowed by the gauges, according to their specifications (Table 2).

### 2.5. Analytical Model

Next, an analytical model of the proposed design is included for a better understanding of the simulation results.

The proposed design is a variation of an elastic body with crossbeams. As the elastic body is symmetric about the X and Y axes, the analysis focused only on one crossbeam.

**Figure 7:**
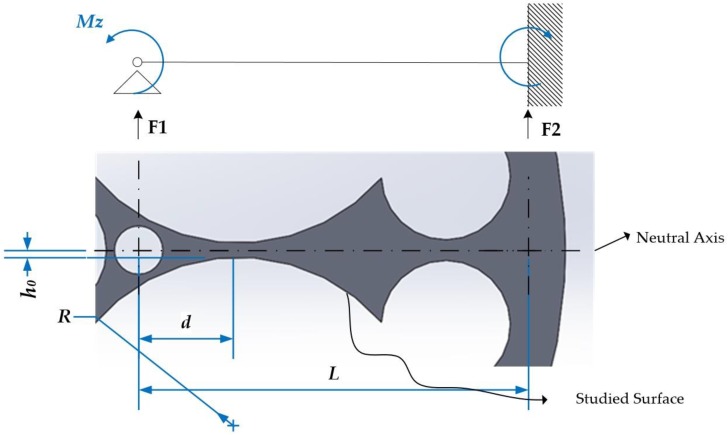
Mechanical model of an elastic body under Mz.

Under the effect of a moment, $M_{z}$, the curved crossbeam will deform, as shown in Figure 4. That means, the curved crossbeam is under a load of flexion along it. Due to the ties, the external ring of the elastic body is rigid, so the curved crossbeam is embedded by its right end. In its left end, where the torque is applied, the curved crossbeam is free to rotate around the Z axis, but it does not have the freedom to shift since its union to the central node prevents shift. Thus, it is considered like a crossbeam embedded at one end and supported on the other end, as it is shown in Figure 7.

Crossbeams are commonly undetermined static structures. Therefore, it is necessary to use the principle of overlapping in order to determinate their reactions to external loads.

**Figure 8:**
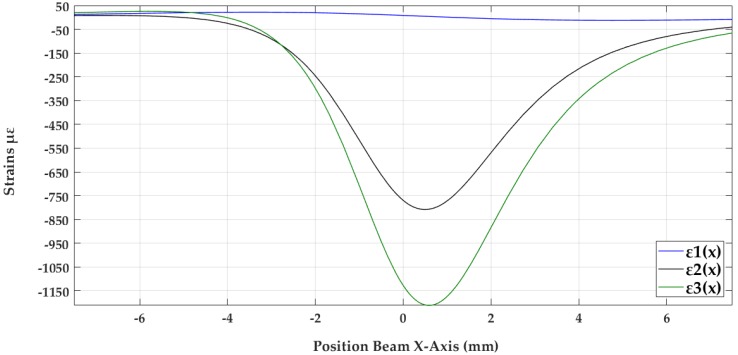
Behavior strain of the elastic bodies with geometries 6a, 6d and 6d optimized.

Once the reactions are defined, the bending moment, $M_{f}\left(x\right)$, acting on the curved crossbeam can be determined. This bending moment depends on the x location along the curved crossbeam. The stress, $\sigma_{x}$, and the strain, $\varepsilon_{x}$, on the surface of the curved crossbeam under the action of a torque ($M_{z}$) can be obtained as follows:

(4) $$\sigma_{x}=\frac{M_{f}\left(x\right)\cdot C\left(x\right)}{I\left(x\right)}=\frac{3\cdot M_{z}\cdot \left(3\cdot x+L-3\cdot d\right)}{16\cdot b\cdot L}\cdot \frac{1}{\left(h_{0}+R-\sqrt{R^{2}-x^{2}}\right)},$$

(5) $$\varepsilon_{x}=\frac{M_{f}\left(x\right)\cdot C_{s}\left(x\right)}{E\cdot I\left(x\right)}=\frac{3\cdot M_{z}\cdot \left(3\cdot x+L-3\cdot d\right)}{16\cdot E\cdot b\cdot L}\cdot \frac{1}{\left(h_{0}+R-\sqrt{R^{2}-x^{2}}\right)},$$

where I(x) is the moment of inertia of the cross section and C(x) is the distance from the neutral axis of the curved crossbeam to the studied surface. Both variables are related with the curved geometry of the surface. b is the thickness of the sensor, L is the crossbeam length, R is the radius of the circumference that defines the studied surface, d is the distance from the center of the circumference to the end of the crossbeam, E is the material elasticity module, and $h_{0}$ is the minimum height of the crossbeam. Figure 8 presents two of the cases analyzed by finite elements.

The first curve $\varepsilon_{1}\left(x\right)$, shown in blue in the Figure 8, corresponds with the sensor that has only big size holes, as in Figure 6a, where can be seen in red and blue the compression (down part of the blue curve) and tension (up part of the blue curve) effects on the same face of the crossbeam. The second curve $\varepsilon_{2}\left(x\right)$, shown in black in Figure 8, represents the case similar to Figure 6d, that include other small size holes in order to increase the length of the crossbeam and thus the strain on the narrower zone.

The last curve, $\varepsilon_{3}\left(x\right)$, shown in green in Figure 8, represents an optimizing of the geometry from Figure 6d. The optimized design is achieved by decreasing the parameter b and increasing the parameter R of the proposed sensor.

## 3. Results and Discussion

Following the steps above, the results obtained from the manufacture of three sensors and the steps carried out to achieve them are displayed below.

### 3.1. Design Optimization

The way to increase deformations to the desired value of 1000 με, an acceptable value measured via gauges, was achieved through small design modifications, such as reducing the sensor thickness to 6 mm and verifying its influence using new simulations. The use of finite elements allows us to obtain the optimized design.

During this process, the objective was to homogenize and standardize the size of drills and positions, reducing the number of required tools, while ensuring market viability. For example, a hole of diameter 10.43 mm should be modified to 10.5 mm to facilitate the purchase of the tool at a reduced price.

**Figure 9:**
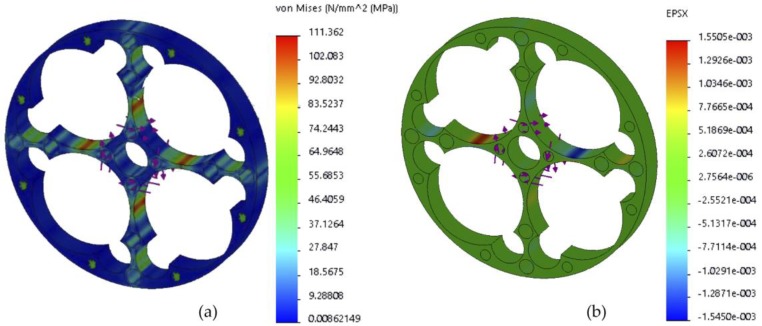
Finite element analysis: (**a**) Stress analysis; (**b**) Strains analysis.

Figure 9 shows the final design of the elastic element of the 1 Nm torque sensor and results after analysis.

The analysis yielded a maximum deformation of 1550.5 με and a maximum Von-Mises stress equal to 111.362 MPa. This value exceeds the 1000 με desirable, but as the strain gauge tends to average the measurement, and considering that its length is greater than the area of maximum strain, it will measure high and medium strain zones, so in the calibration process the value will be slightly reduced, being equally acceptable.

To evaluate the degree of coupling of the sensor, other finite element analyses are carried out considering different load directions. This will have a maximum coupling error of 10%, if forces of 800 N are generated along the direction of the X or Y axis.

An analysis of the transverse sensitivity of the sensor, through strains in the Z direction, indicated that the geometry design is beneficial, as the sensitivity decreases compared with that expected with a geometry composed of flat beams.

Finally, the analysis for the other sensors can be easily made by changing parameters in the initial design. Table 3 presents the maximum strain for the developed sensors.

### 3.2. Analysis of Deviations in Manufacturing

As a prior analysis to the manufacture of the elastic element, the possible effects on production with a drilling machine have been considered, since this device is very economical, albeit not very precise. For this reason, several variations were provided in the 3D model of the final design, with the intention of observing, through finite element analysis, the impact of these deviations on the result of strains and torque measurement.

On the other hand, possible measurement errors are classified into three types according to the resulting effects: offset, gain, and nonlinearity. If not excessive, offset errors are easy to correct, using software for example, and are mainly due to the electronic or mechanical setup of the sensor. Gain errors can be adjusted by weighting with external resistors to the bridge through the amplification system. The most serious error is non-linearity, because it cannot be easily corrected after the measurements.

**Figure 10:**
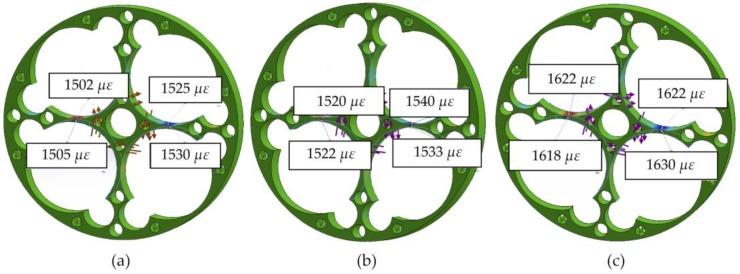
Strain variation in accordance with tolerance: (**a**) Location, (**b**) Size, and (**c**) Flatness.

Following, possible machining deviations are analyzed because they could change strains and, therefore, induce measurement errors. It is important to emphasize that variations in strains $\varepsilon_{3}$ and $\varepsilon_{2}$ will have considerable influence since the final output voltage is linearly proportional to their change., equation (3). As an example, Figure 10 shows the result of the strain analysis in the direction of the X axis, before the variations in size or position of the 8 mm drill, 23.5 mm drill, and the sensor width.

A comparison of the theoretical voltage output of the Wheatstone bridge with the ideal design is shown in Table 4. In conclusion, the relation voltage/torque may slightly change the offset and/or the slope, but they keep very good linearity, corroborating the good properties of the selected material.

Through these results, it can be corroborated that the use of imprecise coarse operations with equipment, such as a drill for boring holes and a conventional saw for cutting thickness, has a very small effect on the results of measurement of the torque. Nevertheless, manufacturing tolerances will have to comply with the accepted limits to avoid those fluctuations and to guarantee the right assembly of the sensor in the final device.

### 3.3. Dynamic Properties of the Sensor

The identification of dynamic properties of the sensor was made with a finite elements model in SolidWorks Simulation.

A modal analysis was performed to obtain the dynamic response of the sensor to different input frequencies. The analysis was made in the condition of real work. The first six resonance frequencies are shown in Table 5.

**Figure 11:**
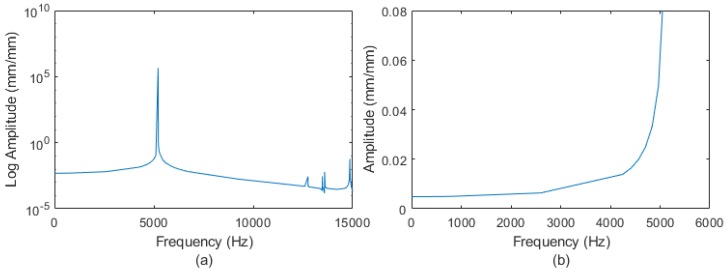
Harmonic response of the diagrams under the measuring of torque Mz: (**a**) Range 0–15,000 Hz (**b**) Range 0–6000 Hz.

In addition, a harmonic analysis was performed to identify and predict the dynamic behavior of the sensor subject to harmonically varying loads. In Figure 11, the diagrams of the elastic deformation can be appreciated to verify if the design can withstand high frequency input torques without resonance.

Figure 11a displays the frequency response and the first six resonance frequencies. Figure 11b shows the frequency range between 0 and 5200 Hz, from which it can be concluded that the sensor bandwidth is between 0 to 2000 Hz. In this range, we can obtain a good precision of the results. For higher frequencies up to 4000 Hz, the sensor could be used if it is calibrated in depth. After this zone, control problems could appear due to the approach of the resonance frequency.

**Figure 12:**
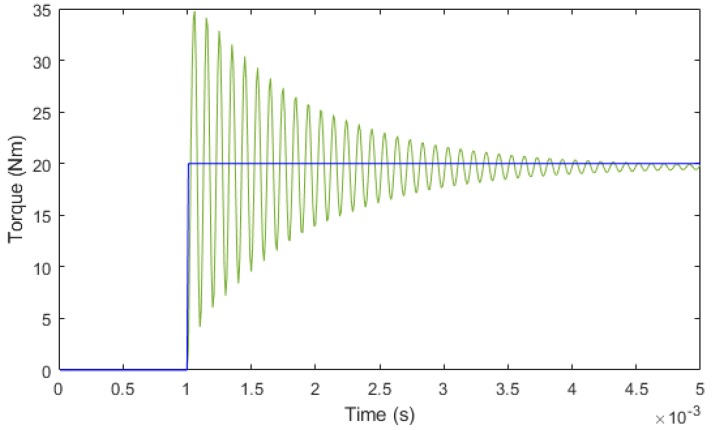
Time response of the sensor with a step input of 20 Nm.

The simulation of a step input of 20 Nm was performed and the results before filtering are represented in Figure 12. It can be seen that the establishment time is 0.02 ms.

### 3.4. Manufacturing Process

As the objective of the work is to reduce the manufacturing cost of the sensor, low-cost manufacturing operations, sawing, and drilling were tested. For this, basic equipment (or variations thereof) was used, as well as other equipment in order to improve the position of drilled holes.

These are:

- Conventional saw for metals (model SABI-SM).
- Conventional column drill without manual control of the displacement of the bench where the part is attached (model INCO-3Z).
- Conventional column drill with manual control of the displacement of the bench where part is attached (model Optium BF 20 Vario).
- Milling machine with position control in its bench (model Kondia 500).

The base material was a 7075-T6 cylindrical billet with a diameter of 65 mm. A 6 mm thick section was cut from the billet using a manual saw. Sawing is a very economical and simple operation, and although it is not a precise method to obtain a flat face, this lack of precision has no effect, since the surface is mostly eliminated by drilling operations, as may be seen in the sensors shown in Figure 6.

For the most economical way to manufacture the sensor, a conventional drilling machine without position control would be used. However, lack of precise control in the positioning of drilling centers causes breakage in the resulting walls, as these are designed to be very thin due to the high sensor-sensitivity requirement.

**Figure 13:**
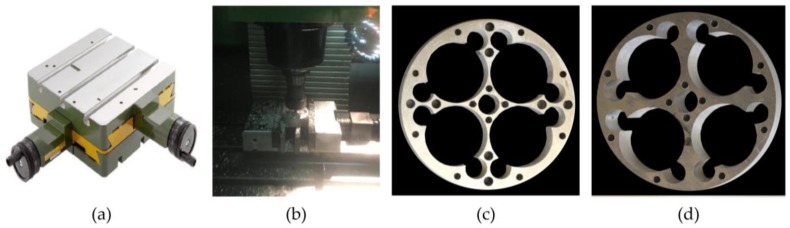
Improvements in manufacturing (**a**) Manual control bench; (**b**) Computerized numerical control (CNC) milling machine; (**c**) Sensor 1 Nm; (**d**) Sensor 20 Nm.

As a second economical manufacturing alternative, a drill with position control in the bedplate was used (Figure 13a), enabling manufacturing that prevents breakages but with a slightly increased cost. This type of machine may be found in most laboratories, or in the case of subcontracting the service, it has a very low machine hourly rate.

As a final alternative, and without discarding the idea of quick, simple operations, if greater automation is preferred, a CNC machine (computerized numerical control) may be used or subcontracted. Due to the type of operations to be carried out, it is not even necessary to prepare a numerical control (NC) program; the “Teach in” mode may be used directly to drill holes. This last alternative also has the advantage of a reduction of total production time and, therefore, costs; however, the higher hourly rate for this type of machine causes the final cost to increase slightly.

Testing of the milling process was satisfactory. Manufacturing times were low, and as the design and manufacture of the sensor were parameterized, the geometries for the other sensors were quickly obtained. The modifications were verified through finite element analysis and the manufacture of the 5 and 20 Nm sensors was carried out easily.

Figure 13a shows a small manual control bench, the manufacturing process using a CNC milling machine with simple operations (Figure 13b), and the result obtained for two of the three sensors (Figure 13c,d).

A postprocess dimensional check on the three sensors showed that in no case are subsequent reaming operations required to improve the surface and dimensional finish of the holes, which has a favorable effect on cost. The final weight of the elastic elements is 17.34, 29.14, and 31.10 gr for the 1, 5, and 20 Nm sensors, respectively, proving that they fulfil the requirement of being lightweight.

### 3.5. Calibration Procedure

To obtain the behavior curve of the sensor, voltage vs. torque, a calibration process is required. In this process, a discrete set of weights will be applied to the sensor in order to obtain, through an electronic circuit, the corresponding variations in voltage. This procedure must be precise, economical and easy to reproduce.

#### 3.5.1. Calibration Bench

**Figure 14:**
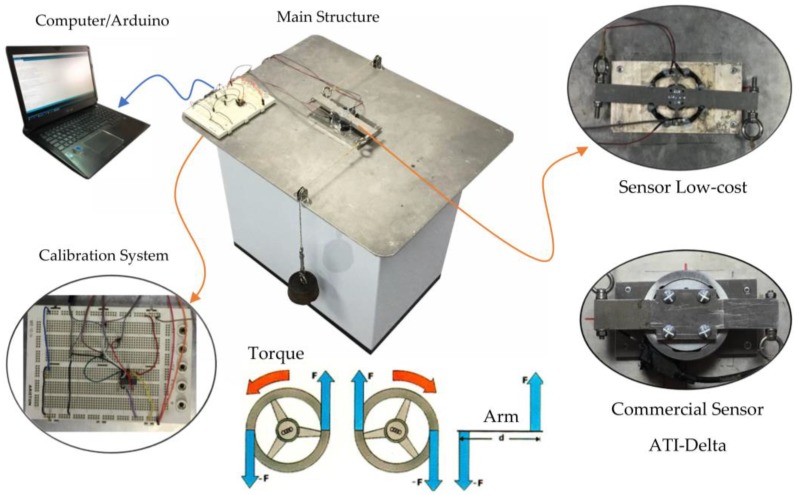
Calibration bench.

Figure 14 shows the calibration bench developed to produce exclusively pure torque in order to ensure that only deformation resulting from the torque is detected by the strain gauges. This bench enabled static calibration of the sensor and was comprised of a main structure and an electronic calibration system.

The main structure was comprised of a metal base that supported the sensor and a lever and pulley symmetric system, which allowed two forces to be applied in opposite directions in order to generate pure torque, as outlined in Figure 14.

The electronic calibration system consisted of a signal amplifier, data acquisition system, and personal computer. 

The gauges were configured on a Wheatstone bridge, the output of which was routed to a Texas Instruments INA125P instrumentation amplifier. The calibration circuit supplied the Wheatstone bridge with a reference voltage of 2.5 V and 5 V for the bridges using gauges of 120 Ω and 350 Ω, respectively. This different voltage was due to the fact that, in the case of the sensor with gauges of 120 Ω, the INA125P was not capable of supplying the required current to provide a reference voltage of 5 V. In the case of gauges of 350 Ω, it did not present this effect. The bridge output signal, in mV, was amplified by the INA125P, with an average gain of 607 and 333 for the 1 and 20 Nm sensors, respectively, and routed to the acquisition system.

The INA125P circuit was selected because it is an amplifier specially designed for Wheatstone bridges, containing an internal voltage stabilizer (voltage fluctuations are a potential source of error) and being of low cost. As will be seen later, the results were quite satisfactory. 

For the acquisition system, a simple Arduino Uno board was selected that incorporates a Microchip/Atmel ATmega328P microcontroller with a 10-bit AD converter. The Arduino board performed conversions continuously and transmitted them through the USB output to a personal computer.

The calibration bench was verified through of a commercial sensor ATI-Delta SI-330-30 (Figure 14). A maximum error of 0.23% of full scale was obtained in the torque measurement.

#### 3.5.2. Calibration Procedure

**Figure 15:**
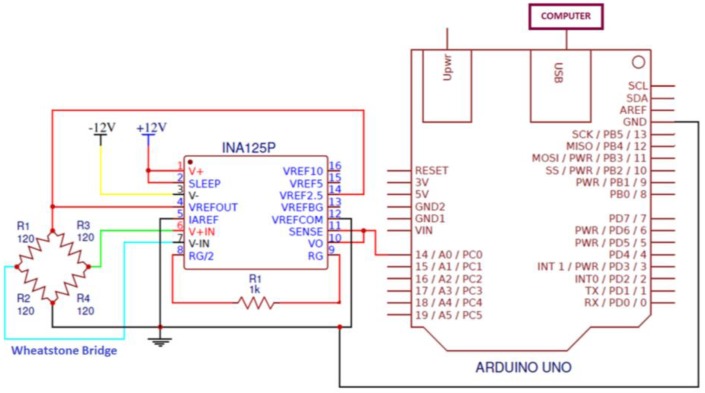
Calibration circuit.

Prior to calibration, the gauges were carefully attached to the elastic element. Afterwards, the complete sensor was installed in the calibration bench. The Wheatstone bridge circuit, amplification system, and Arduino Uno controller were connected, as shown in Figure 15.

**Figure 16:**
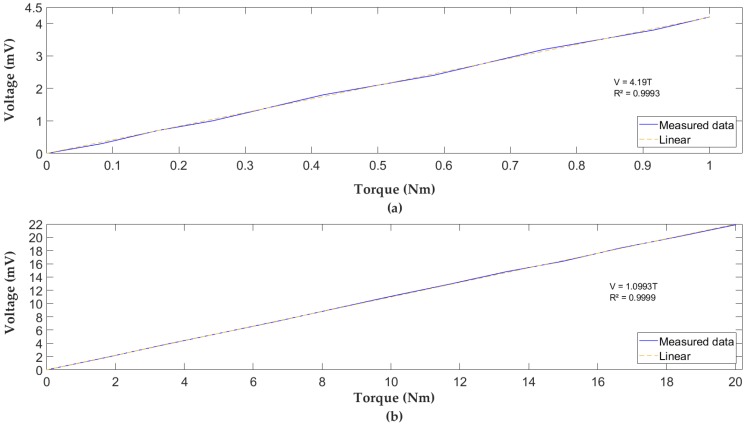
Graph bridge output voltage vs. applied torque: (**a**) 1 Nm Sensor, (**b**) 20 Nm.

Once the connections had been established, calibration was carried out by changing the weights supported by the sensor and capturing the output variations. For example, Figure 16 shows the graphs of the output voltage of the Wheatstone bridge versus the applied torque for the 1 and 20 Nm sensors.

For the 1 Nm sensor, loads of 0 to 0.6 Kg were applied with an increment of 0.05 Kg. In the case of the 20 Nm sensor, loads of 0 to 12 Kg were applied, with increments of 1 Kg. Both sensors had very good linearity (linear regression coefficient *R*^2^ near to 1). Obtaining the calibration line for both sensors enabled comparison of the calculated and applied torque. The results of the calibration of the sensors presented excellent results. These are shown in Table 6. It can be seen that the 1 Nm sensor has a linearity and measurement error more than those of the 20 Nm sensors. Instead, it has more sensitivity, better resolution, and better torsional stiffness.

Table 7 allows one to compare the values of sensitivity and torsional stiffness with respect to a Hub-Sprocket sensor (four flat beams) with similar dimensions and features. The geometry of curved beams presents a better performance for the sensor of 1 Nm and is quite similar for the 20 Nm.

Although the calibration process is economical and enables good results, it is very susceptible to typical variations, such as, electronic assembly sensitivity, mechanical friction of the pulley system at low weights, etc. Therefore, it is advisable to use proper components in order to prevent these problems.

### 3.6. Manufacturing Costs

The cost analysis for the manufacture of the sensor, by drilling, milling, and an external CNC company [26], are shown in Table 8.

Labor costs correspond to the preparation of the machine tool. Machinery costs correspond to manufacturing costs, machining time, and tools used. It should be noted that the first two processes incur design costs, equal and constant for both, and, therefore, are not included in the calculation.

It should be taken into account that the manufacturing cost may vary depending on the country and many other factors. The prices shown are real in our context but indicative in other cases. On the other hand, the production of the type of sensors presented in this article is oriented to prototype or small series. In the case of a large series, an automate production will lower the cost.

A sensor designed with a similar size Hub-Sprocket was developed in our facilities with a real cost of €183.6, which reinforces our hypothesis that simple operations reduce the manufacturing cost.

The cost of milling is twice the cost of drilling. Nevertheless, both costs in this proposal are low compared to that of commercial sensors. Development of the sensor is economical, thus, manufacture by milling may be defined as “low cost” and manufacture by drilling as “ultra-low cost”.

The total cost of the sensor, including manufactured parts, gauges, amplification electronics, and digital microcontroller system would be below €100.

## 4. Conclusions

A very important part of the cost of torque sensors is related to manufacturing (machining). Therefore, a more affordable and simpler form of production has been sought. All torque sensors currently available on the market have flat beams (Hub-Sprocket type) or complex geometries requiring more laborious operations. However, in this proposal, curved crossbeams were chosen in order to facilitate machine manufacture.

Drilling is much more affordable than milling. Thus, if the geometry of the sensor is adapted to be achievable with a drill, standard tools should be used to reduce costs drastically. However, for geometries with very thin wall thicknesses, positioning control is required, normally on the X and Y axes where drilling operations are carried out. In these cases, inclusion of a manual position control would suffice.

Finite element analysis of admissible geometric variations was crucial for the selection of machining equipment and operations that truly facilitated a considerable reduction of costs.

Moreover, the proposed calibration method enables manufactured sensors to be characterized and conditioned for operation in an easily reproducible manner.

It should also be noted that development of future sensors shall not exceed the values shown, since having parameterized the design, modification of dimensions and manufacturing of new elastic elements for different loads will be a much faster process.

The extension of the low-cost solution to multiaxis sensors could be possible if the sensor was made by an assembled structure of parts and each part was made by simple machining operations. This would allow for a reduction in the cost compared to monolithic sensors with very complex geometries.

In summary, a torque sensor has been developed at a cost much lower than those commercially available and with fully operational results. Any laboratory or research center with simple machines can develop their own customized sensor to be used in their prototypes or small series, with a more affordable price.

If the sensors are going to be commercialized, other associated costs should be kept in mind, such as costs related to packaging, advertisement, software, profit, etc. They are outside the scope of this article.

## Figures and Tables

**Table 1:** Mechanical Properties: Aluminum 7075-T6.

Property	Density (g/cm^3^)	Young’s Modulus (MPa)	Poisson’s Ratio	Yield Strength Sy (MPa)	Ultimate Strength Su (MPa)
Value	2.80	71.7	0.33	503	572

**Table 2:** Strain gauge specifications.

Parameters	Contents
Gauge Factor	2 ± 1%
Gauge Resistance	120 ± 0.35%/350 ± 0.35% Ω
Gauge Size	6 mm × 2 mm
Minimum Radius of Curvature	10 mm

**Table 3:** Strain of the sensors.

Torque Sensor	Maximum Strain
1 Nm	1550 με
5 Nm	2293 με
20 Nm	2384 με

**Table 4:** Voltage variation in accordance with manufacturing deviation.

Case	Machining Deviation	Nominal Dimension	Voltage Output Variation
Figure 10a	Offset of +0.5 mm	Hole of Ø 8 mm located in the beams	−2%
Figure 10b	Increase +0.5 mm in Ø	Big Hole of Ø 23.5	−1%
Figure 10c	1° of surface inclination	flatness (differences in thickness)	+4%

**Table 5:** Resonance Frequencies.

Mode	1	2	3	4	5	6
Responding Frequency (Hz)	5214.4	5668.9	12,752	12,773	13,478	13,589

**Table 6:** Calibration results.

Sensor	Linearity Error (%F.S.)	Measurement Error (%F.S.)	Strain με	Sensitivity (mV/Nm)	Resolution (Nm)	Hysteresis Error (%F.S.)	Torsional Stiffness (10^6^ Nm)
1 Nm	1.27	1.92	1004	4.19	0.002	3.54	3.57
20 Nm	0.61	1	2207	1.09	0.02	4.64	2.15

**Table 7:** Sensitivity and torsional rigidity for sensor type Hub-Sprocket.

Sensor	Sensitivity (mV/Nm)	Torsional Stiffness (10^6^ Nm)
1 Nm	2.85	4.14
20 Nm	0.99	2.56

**Table 8:** Manufacturing costs per machine type.

Concept	Labor Costs	Total Machinery Costs	Total Cost
Drilling Sensor	€4.00	€20.00	€24.00
Milling Sensor	€10.00	€52.50	€62.50
CNC Milling sensor	External Manufacturing		€165.23
